# Fabrication and Characterization of Electrospun DegraPol^®^ Tubes Releasing TIMP-1 Protein to Modulate Tendon Healing

**DOI:** 10.3390/ma18030665

**Published:** 2025-02-03

**Authors:** Julia Rieber, Roger Khalid Niederhauser, Pietro Giovanoli, Johanna Buschmann

**Affiliations:** Department of Plastic Surgery and Hand Surgery, University Hospital Zurich, Sternwartstrasse 14, 8091 Zurich, Switzerland; julia.rieber@usz.ch (J.R.); roger.niederhauser@usz.ch (R.K.N.); pietro.giovanoli@usz.ch (P.G.)

**Keywords:** proliferation, gene expression, water contact angle, electrospinning, release kinetics, Fourier transformed infrared spectroscopy (FTIR)

## Abstract

Background: Tendon rupture repair can result from fibrotic scar formation through imbalanced ECM deposition during remodeling. The tissue inhibitors of matrix metalloprotease (TIMPs) not only decrease ECM degradation, regulated by matrix metalloproteases (MMPs), but also restrict TGF-β1 activation and thus diminish fibrosis. Methods: Rabbit tenocytes (rbTenocytes) and rabbit adipose-derived stem cells (rbASCs) were cultivated under different TIMP-1 concentrations. Proliferation and gene expression were assessed. TIMP-1 was incorporated into emulsion electrospun DegraPol^®^ (DP) tubes that were characterized by SEM for fiber thickness, pore size, and wall thickness. Static and dynamic water contact angles, FTIR spectra, and TIMP-1 release kinetics were determined. Results: While the proliferation of rbTenocytes and rbACS was not affected by TIMP-1 supplementation in vitro, the gene expression of *Col1A1* was increased in rbTenocytes, the gene expression of *ki67* was increased in both cell types, the gene expression of *tenomodulin* was increased in both cell types at 100 ng/mL TIMP-1, and alkaline phosphatase expression *ALP* rose significantly in rbASCs. Electrospun TIMP-1/DP fibers had a ~5 μm diameter, a ~10 μm pore size, and a mesh thickness of ~200 μm. TIMP-1/DP meshes were more hydrophilic than pure DP meshes. TIMP-1 was released from the meshes with a sustained release of up to 7 days. Conclusions: TIMP-1/DP tubes may be used to modulate the fibrotic tissue reaction when applied around conventionally sutured tendon ruptures.

## 1. Introduction

Tendon rupture repair is still challenging because tendons have a low cell density and the tenocytes are metabolically not very active [1]. Moreover, tendon tissue is practically avascular, and tenocytes mostly receive the necessary oxygen and nutrients via diffusion [2]. In turn, tendons heal very slowly, and tendon ruptures very often result in inferior mechanical stability caused by fibrotic scar tissue—in the worst cases, this leads to re-rupture [3,4]. Therefore, agents that modulate and support tendon healing are currently the focus of many orthopedic research groups [5,6,7,8,9,10].

During the last decades, the application of growth factors has gained growing attention [11]. After tendon rupture, platelets secrete growth factors with distinct timed dynamics [12], such as platelet-derived growth factor-BB (PDGF-BB), insulin-like growth factor-1 (IGF-1), transforming growth factor-beta (TGF-β), or basic fibroblast growth factor (bFGF), among others [13]. What nature provides during natural tendon healing may be modulated by accentuating certain effects to accelerate the tendon healing and in turn may enable patients to return to physical activity earlier. For example, implant materials with immobilized growth and differentiation factor-7 (GDF-7) [14] or materials that release growth factors slowly to the ruptured and repaired tendon [15] may enhance the healing process to achieve higher tensile strength at earlier time points post-operation, as exemplified for PDGF-BB in a rabbit Achilles tendon full-transection model [16].

Tendon healing consists of three overlapping phases, with an initial inflammation, a subsequent cellular phase, and a final consolidation stage [2]. During the first inflammatory stage, a lot of inflammatory cells, such as neutrophils and macrophages, enter the wound site, removing the debris of the damaged tendon cells. Then, during the second cellular phase, intrinsic and extrinsic tenoblasts occur, proliferate, and build up an initial collagen III-containing template of a new extracellular matrix, which is still disorganized in this state. During the third remodeling and consolidation stage, there is a realignment of fibers in the direction of loading and gradual replacement of collagen III by the predominant type I collagen [1]. Hence, the remodeling of the extracellular matrix (ECM) is a key event during tendon healing, as a proper and stably aligned fiber network has to be rebuilt, while cellular and fiber debris must be appropriately degraded and removed. Besides force-induced remodeling processes [17], enzymatic ECM degradation by matrix metalloproteases (MMPs) [18] in balance with their inhibitors (tissue inhibitors of MMPs—TIMPs) [19] plays a fundamental role. Hence, bioactive implant materials that modulate the delicate balance of MMPs and TIMPs may be useful in studying the healing process of tendons and serve as triggers for the preclinical models of fibrotic healing versus regenerative (non-fibrotic) healing. Besides its inhibiting effect for MMP-1, MMP-2, and MMP-9, TIMP-1 has been reported to inhibit ADAMs (a disintegrin and metalloproteinases) [20,21]. ADAMs are a family of surface proteins that are involved in proteoglycan cleavage that is important not only in tendons [22], but also in cleaving proteins close to the surface and/or overexpressed in endothelial cells, liver cells, and monocyte macrophages in hepatocellular carcinoma [23]. Along with these proteinase-inhibiting effects, TIMP-1 can bind to several cell surface proteins, such as CD82, CD63, and LRP1, respectively, particularly when TIMP-1 concentrations exceed MMP concentrations. In other words, the free TIMP-1 protein that is not complexed to any MMPs may act as a cytokine and trigger different signaling pathways via cell surface receptor binding, thereby activating cell growth, proliferation, and anti-apoptosis [21], processes that promote tendon regeneration and potentially accelerate the healing process.

Against this background, we considered TIMP-1 as an interesting bioactive protein that may modulate tendon rupture repair by enhancing proliferation and anti-apoptosis and may, therefore, be interesting for applications during the early phase of tendon healing. As a 28 kDa protein, TIMP-1 has a molecular weight that is suitable for emulsion electrospinning, a method previously established in our research group [15,24]. Therefore, we fabricated an emulsion electrospun biodegradable and biocompatible DegraPol^®^ fiber mesh [25,26], with TIMP-1 protein incorporated, and characterized the new material by the assessment of fiber diameter, pore size, and wall thickness using scanning electron microscopy (SEM). We also established Fourier Transform Infrared (FTIR) spectra and static as well as dynamic water contact angles, respectively. Release kinetics were assessed, as was the impact of TIMP-1 on the gene expression of rabbit Achilles tendon tenocytes (rbTenocytes) and rabbit adipose-derived stem cells (rbASCs) in vitro. The typical tendon-related marker gene *tenomodulin*, proliferation marker *ki67*, and ECM marker *Col1A1*, encoding collagen I protein, were assessed, as was *alkaline phosphatase* (a typical early marker during osteogenesis). The latter was analyzed because TIMP-1 has been reported to act beneficially at the tendon–bone interface and the enthesis too [27].

Like this, we present a novel tubular implant material to be applied during tendon rupture surgery around a conventionally sutured tendon to promote mid-substance tendon tissue healing or potentially to improve the healing at the enthesis region when placed at the *calcaneus*-Achilles tendon transition [28]. To the best of our knowledge, such emulsion electrospun biodegradable and biocompatible DP random fiber meshes with incorporated TIMP-1 protein have not been fabricated to date.

## 2. Materials and Methods

### 2.1. The Polymer DegraPol^®^ (DP)

For the synthesis of DP, 25 wt% of poly (3-(R-hydroxybutyrate)-co-(ε-caprolactone)-diol (Mn = 2824 g mol^−1^) and 75 wt% of poly(ε-caprolactone)-diol-co-glycolide (15 mol% glycolide, 85 mol% ε-caprolactone) (Mn = 1000 g mol^−1^) were dissolved in 1,4-dioxane and dried until water content was below 20 ppm. The solution was cooled and a stoichiometric amount of 2,2,4-trimethylhexane-diisocyanate (TMDI) was added. After one day, dibutyltin dilaurate (20 ppm) was added three times within 1 d in order to reach a molecular weight of 100–110 kDa. The polymer was precipitated in cooled hexane isomers and purified with chloroform and a silica gel 60 column (Fluka, Basel, Switzerland), followed by precipitation in cooled ethanol [24,25].

### 2.2. Incorporation of the Growth Factor TIMP-1 into DP

The solutions were prepared 1 to 3 days before electrospinning. For each scaffold, a polyethylene glycol (PEG) (35 kDa, Sigma-Aldrich, Steinheim, Germany #81310) solution was prepared by adding 1.5 g of PEG and 3.5 g of chloroform (Sigma-Aldrich, Germany, #132950). The DP solution was produced by adding 0.6 g of DP powder, 3.52 g chloroform, and 0.88 g of 1,1,1,3,3,3-Hexafluoro-2-propanol (HFP, Sigma-Aldrich, Germany, #105228) into a glass with screw cap. For the incorporation of recombinant human TIMP-1 (PeproTech, Boston, MA, USA, #100-11-100UG), a total amount of 8 µg TIMP-1 dissolved in 400 µL phosphate-buffered solution (PBS, BioConcept, Allschwil, Switzerland, #3-05F39-I) containing rabbit serum albumin (RSA) (10 µg/mL TIMP-1 and 0.25% RSA in PBS) was added dropwise to the DP solution while stirring for 5 min at 500 rpm. The solution was shortly mixed on the vortex and further emulsified in an ultrasonic bath for 15 min. The emulsion was filled in a 5 mL glass syringe (Huberlab, Aesch, Switzerland, # 3.7102.33) and used immediately for electrospinning. As this protocol for water-in-oil emulsion with TIMP-1 was the same as used previously for PDGF-BB [24] and IGF-1 [15] incorporation, a homogenous distribution of water droplets containing TIMP-1 within the DP fibers can be assumed.

### 2.3. Electrospinning

The scaffolds used in this study were fabricated using electrospinning with a custom-designed apparatus. The system consisted of a DC high-voltage power supply (Glassman High Voltage Inc., High Bridge, NJ, USA), with a needle holder and transporter device. The syringe was connected to a blunt-ended spinning head via a Teflon hose, which delivered the polymer solution into a stainless steel needle (1 mm inner diameter and 0.3 mm wall thickness, Angst & Pfister AG, Zürich, Switzerland) made of stainless steel (Angst & Pfister AG, Zürich, Switzerland). A metal rod with a length of 55 cm was mounted to the rotary motor (the Euro Star B rotary motor, IKA Labortechnik, Staufen im Breisgau, Germany) and served as a collector. The electrospinning parameters for fabricating the tubular scaffolds included a flow rate of 1 mL/h, a working distance of 19.5 cm between the needle tip and the metal rod, and an applied voltage of 12.5 kV. The collector rotated at 500 rpm, while the needle transporting the polymer solution moved laterally in a 20 cm range to ensure uniform random fiber deposition. The process was conducted under controlled temperature (22–23 °C), with humidity levels between 25% and 35%. Initially, a layer of polyethylene glycol (PEG) was deposited onto the metal rod to facilitate the detachment of the scaffold. Subsequently, layers of DP or DP combined with TIMP-1 were electrospun onto the collector. After detachment with 50% ethanol, the tubes were stored in a desiccator. After drying for three days to one week, the tubes were stored at 4 °C.

### 2.4. Scanning Electron Microscopy (SEM)

For each scaffold tube, the inner and outer surfaces as well as cross-sections of the tubes were examined. Small sections of the tubes were mounted onto SEM carriers using conductive double-sided adhesive tape coating and imaging was performed with equipment maintained by the Center for Microscopy and Image Analysis, University of Zurich, Switzerland. The samples were coated with a 10 nm platinum layer using a sputter coater (Safematic CCU-010, Jena, Germany). Imaging was performed with a Zeiss Gemini SEM 450, Zeiss, Jena, Germany, at an accelerating voltage of 5 kV. The images were captured at a magnification of 500× with a brightness setting of 49%, using the secondary electron detector. Fiber diameters and tube wall thicknesses were analyzed using the ImageJ software (version 1.53e/Java 1.8.0_172, 64-bit) based on the scale bar from the microscope images. To measure fiber or pore dimensions, a diagonal line was drawn across each SEM image, and all the fibers or pores intersecting the line were measured. For the standardization of the wall thickness, the scheme of a clock was used and SEM pictures were taken at 2, 4, 6, 8, 10, and 12 o’clock, respectively. From each image, there were three random measurements. Per the SEM image, a diagonal line was drawn and all the fibers and pores crossed by the line were measured.

### 2.5. Fourier Transform Infrared Spectrometry (FTIR)

FTIR spectroscopy was performed using a Varian 640 FTIR spectrometer equipped with a Golden Gate diamond ATR unit that is equipped with temperature control. Spectra were recorded across a wavenumber range of 600–4000 cm^−1^, with a resolution of 4 cm^−1^. Each spectrum was generated by averaging 64 scans. For comparative analysis, the ratio between the C=O peak at 1720 cm^−1^ and the C–O peak at 1175 cm^−1^ was calculated. To visually present the data, normalization was performed with respect to the C=O peak at 1720 cm^−1^. The study included the assessments of DP powder, pure DP tubes, and DP tubes with various emulsions. PEG was also analyzed to identify potential contamination. The characteristic bonds were identified by comparing the observed peaks with an IR spectrum reference table (Merck KGaA, Darmstadt, Germany).

### 2.6. Static and Dynamic Water Contact Angles

The water contact angle (WCA) was evaluated for both the pure DP tubes and DP emulsion tubes containing TIMP-1, respectively [29]. To prepare the samples, the tubes were carefully cut open using a scalpel and placed on a glass secured with double-sided adhesive tape. WCA was assessed for both the inner and outer surfaces. The WCAs were measured using a goniometer coupled to an IDS uEye camera, instrument OCA 35 Dataphysics, Berlin, Germany. Droplets with a volume of 5 µL were dispensed onto the surface using a 1 mL syringe filled with Milli-Q water. The analyzer directly recorded the left and right WCAs formed by the droplet, and the mean of these measurements was calculated to define the water contact angle. Each sample underwent at least three measurements.

Dynamic WCAs were also measured using the same goniometer. The advancing and receding water contact angles were determined by incrementally adding or removing water from an initial 5 µL droplet at a rate of 15 µL/min. The measurements of the water contact angle were recorded every second over a period of one minute. Water contact angle hysteresis was calculated by subtracting the receding angle from the advancing angle [30].

### 2.7. Release Kinetics of TIMP-1 from Electrospun Mesh

To assess the kinetics of TIMP-1 release from the DP tubes, three segments of 0.5 cm in length were taken—one from each end and one from the middle of each tube—and placed into separate low-binding microtubes (Eppendorf, Flaach, Switzerland) (n = 3 tubes and n = 3 pieces). The tube had a density of 21 mg/cm^2^, or 21 mg/100 mm^2^, and each segment had a tube diameter of 4 mm and furthermore, a height of 5 mm (0.5 cm). The segment weight was calculated with m = П A = 21 mg/ 100 mm^2^ 5 mm П 4 mm = 13.2 mg, where m is the mass in mg, ρ the density in mg/100 mm^2^, and A the area in mm^2^. We roughly estimated the TIMP-1 content per tube as one-third of the total loading (8/3 μg per tube). Each tube was sectioned into 30 equal small segments for the release kinetics, resulting in approximately 89 ng per tube segment.

Each low-binding microtube was filled with 500 µL of 0.1% RSA in PBS, which served as the release medium. The samples were incubated at 37 °C with shaking at 300 rpm. At specified time points, the medium was collected into labeled microtubes, and fresh release medium was added to the low-binding tubes. The collected release samples were stored at −20 °C until analysis.

The release of TIMP-1 was quantified using the Detergent-compatible colorimetric Assay kit (DC Protein assay, (Bio Rad, Cressier, Switzerland, Nr. 5000111)) in accordance with the manufacturer’s instructions. Absorbance measurements were performed using an ELISA Microplate Reader (BioTek Cytation/5 imaging reader, Agilent Technologies, Basel, Switzerland) at a wavelength of 750 nm. The results were expressed as a cumulative release over time in the percentage of highest concentration at day 7. After completing the incubation process, the scaffolds were stored in the remaining PBS solution at −20 °C.

### 2.8. Rabbit Tenocyte and ASC Culture, Alamar Blue Assay, and Quantitative Real-Time PCR

RbTenocytes isolated from the Achilles tendons of three New Zealand White rabbits were used. The rbTenocytes were thawed and resuspended in culture medium (Ham’s F12 (biowest, Nuaillé, France #L0135-500) with 10% FBS (biowest, Bradenton, South America, #S1830-500), 1% Penicillin/Streptomycin (Sigma, Israel, #P4333-100ML), and 1% glutamax (Life Technologies, Carlsbad, CA, USA, #35050-038)). RbTenocytes from passages 2 (P2) were used. Moreover, rabbit stem cells derived from abdominal adipose tissue (ASCs = adipose-derived stem cells) from three New Zealand White rabbits, where P3–4, were utilized. The impact of supplementation of culture medium by recombinant human TIMP-1 at concentrations of 0, 1, 10, and 100 ng/mL were tested for both rbTenocyte and rbASC culture, respectively.

On days 1 and 3 of cell culture, cell proliferation was measured using the alamarBlue™ cell viability assay (Thermo Fisher Scientific, Waltham, MA, USA). A total of 350 cells per well were seeded in technical quadruplicates in 100 µL of culture medium into a 96-well plate (TPP, #92096) and incubated at 37 °C in a 5% CO_2_ environment. To prevent dehydration, empty wells were filled with PBS. The alamarBlue™ solution was diluted 1:10 in the culture medium and incubated with cells for 4 h. Fluorescence was measured with excitation wavelength at 530 nm and emission wavelength at 590 nm using a Microplate Reader (BioTek Cytation/5 imaging reader) [31]. The percentage of increase was calculated as follows: (cell number at day 3/cell number at day 1) − 1) 100%.

In order to determine the effect of TIMP-1 on the gene expression of rbTenocytes and rbASCs, the cells were seeded into 6-well plates (Sigma, Israel, #SIAL0516, growth area per well: 9.6 cm^2^) with a density of 2 × 10^5^ cells/ well in 2 mL of culture medium. The cells were allowed to attach overnight. The desired TIMP-1 concentration was added to the medium at day 0. The samples were cultured at 37 °C with 5% CO_2_ and collected after three days. Cells from three rabbit donors were used and qPCRs were performed in triplicates. At the respective time point, total RNA was isolated using the RNeasy Plus Mini Kit (Qiagen, Hilden, Germany, #74104) with RNase-free DNase treatment (Qiagen, Hilden, Germany, #74104) following the manufacturer’s protocol. The purity and amount of RNA were measured with Nanodrop One (ThermoFisher, Boston, MA, USA, ND-ONE-W). For reverse transcription (RT), 500 ng RNA was investigated in a reaction volume of 20 µL (SuperScript III Reverse Transcriptase, ThermoFisher, Boston, MA, USA, # 18080085; Oligo(dT) 12-18 Primer, Thermo Fisher, # 18418012; RNase Inhibitor, Applied Biosystem; Waltham, MA, USA, # N8080119, dNTP, Invitrogen, Waltham, MA, USA, #18427013) using a compact thermocycler (appliedbiosystems by Thermo Fisher, Singapore, #A37028) scientific. Real-time PCR reactions were performed in technical triplicates with 4 µL of the resulting cDNA (cDNA was diluted 10-fold with water for the analyses in a reaction volume of 20 µL and 180 µL of water ) using the Quant Studio 5 (Applied Biosystems, Waltham, MA, USA, CYT5M) and Fast SYBR Green Master Mix (Thermo Fisher, Vilnius, Lithuania, #4385612). The samples were heated to 95 °C for 3 min, followed by 40 cycles of 95 °C for 3 s and 60 °C for 20 s. All the rabbit primers, listed in Table S1, were synthesized by Microsynth, Balgach, Switzerland. A relative expression analysis was performed using the comparative 2^−∆∆CT^ method with 18S as a reference gene, which was stable over the conditions analyzed. The results are presented as fold change normalized to control, i.e., compared to the averaged samples cultivated without TIMP-1 (normalized to 1) [31].

### 2.9. Statistics

Data were analyzed by GraphPad Prism 10 (Version 10.3.1 GraphPad Software Inc., San Francisco, CA, USA). A Shapiro–Wilk test was used to test for the normal distribution of the data. For comparing two groups, an unpaired *t*-test was used in the case of the normal distribution of data. Otherwise, a Wilcoxon test was applied. For the analysis of more than two groups, the following tests were used: Where normally distributed data were confirmed, a one-way analysis of variance (ANOVA with Tukey’s multiple comparisons test) was used for pairwise comparison or a two-way ANOVA in case different levels were compared. If the data were not following normal distribution, a nonparametric Kruskal–Wallis test was used. *p*-values ≤ 0.05 were considered significant and denoted with (*); for *p* ≤ 0.01 (**); for *p* ≤ 0.001 (***); for *p* ≤ 0.0001 (****); and for non-significant (ns). The calculations of standard curves and other calculations were performed in Excel (Version 2402 Build 16.0.17328.20550, 64 Bit)

## 3. Results

### 3.1. Impact of TIMP-1 on rbTenocyte and rbASC Proliferation

**Figure 1:**
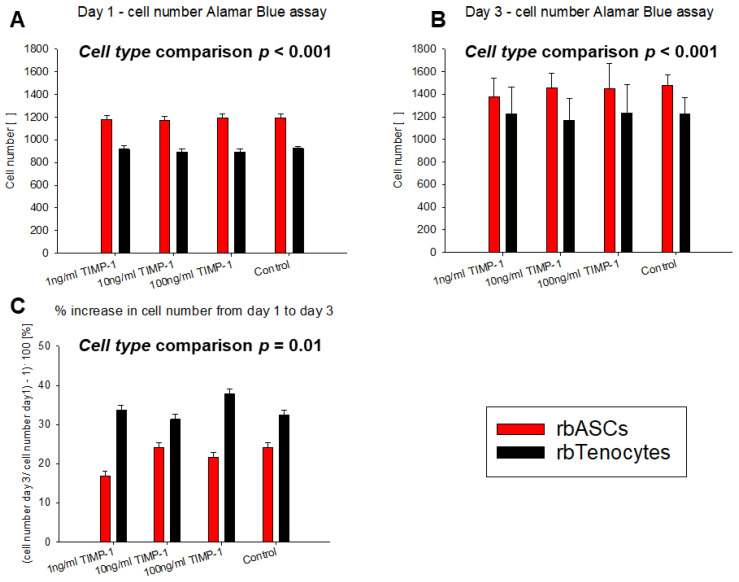
Proliferation of rbASCs (red) and rbTenocytes (black) under TIMP-1 supplementation determined with the Alamar Blue^®^ assay. Absolute cell numbers after 1 day (**A**) and 3 days of culture (**B**) with the same initial cell number. The relative increase in cell numbers in % between day 1 and day 3 (**C**). A two-way ANOVA with the levels of *TIMP-1 concentration* and *cell type* revealed a significant difference between the two cell types, with *p* < 0.001 for (**A**,**B**) and with *p* = 0.01 for (**C**); however, no significant differences were found with respect to level TIMP-1 concentration.

To determine the effect of 1, 10, or 100 ng/mL TIMP-1 supplementation on the culture medium of either rbTenocytes or rbASCs, the Alamar Blue^®^ assay was performed on day 1 and day 3, respectively. Starting with the same initial cell numbers, Figure 1 represents the respective cell numbers obtained after one day of cultivation and after three days as well as the relative increase in cell numbers, given as a percentage of increase between day 1 and day 3 for each cell type. While rbASCs exhibited a higher proliferation rate, with significantly higher cell numbers compared to rbTenocytes on day 1 and day 3 based on a two-way ANOVA with levels of *TIMP-1 concentration* and *cell type*, the percentage of increase in cell numbers was significantly higher for rbTenocytes compared to rbASCs. However, the three different concentrations of TIMP-1 did not have a significant impact on the proliferation of either cell type.

### 3.2. The Influence of TIMP-1 on the Gene Expression of Rabbit Tenocytes and ASCs

**Figure 2:**
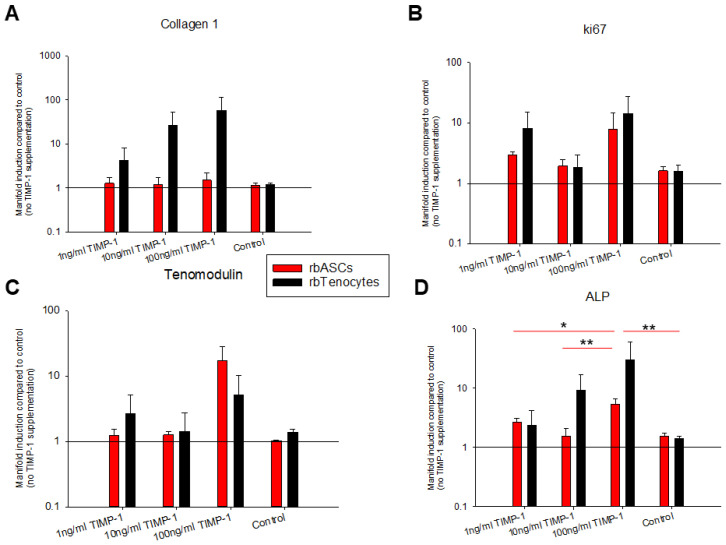
RbASC (red) and rbTenocyte (black) gene expression as mean ± standard error of the mean obtained from three donors for *Col1A1* (**A**), *ki67* (**B**), *tenomodulin* (**C**), and alkaline phosphatase *ALP* (**D**) under the TIMP-1 supplementation of 1, 10, or 100 ng/mL or without TIMP-1 (control). Key: using a Kruskal–Wallis test, the mean of 3 donors was calculated per condition, and inter-group significant difference was attributed to *p* ≤ 0.05 (*) and *p* ≤ 0.01 (**), respectively.

The gene expression for *collagen 1A1* (*Col1A1*), *ki67*, *tenomodulin*, and *ALP* was assessed for rbTenocytes and rbASCs under different TIMP-1 concentrations (1, 10, and 100 ng/mL) and normalized to the control (cells without TIMP-1 supplementation). Figure 2 shows that the *Col1A1* gene expression of rbASCs remained unaffected under TIMP-1 supplementation, while a trend towards a dose–response was found for rbTenocytes: the higher the TIMP-1 concentration, the higher the *Col1A1* gene expression. A two-way ANOVA confirmed a significant difference between the two cell types for *Col1A1* gene expression. As for *ki67*, the two cell types showed similar response towards TIMP-1 supplementation, with elevated gene expression at 1 and 100 ng/mL TIMP-1 and only slightly higher *ki67* levels at 10 ng/mL TIMP-1 when compared to the control. The tendon-specific marker *tenomodulin* was elevated at 1 and 100 ng/mL TIMP-1 in tenocyte culture and at 100 ng/mL TIMP-1 in rbASC culture compared to the control; however, this observed increase was not statistically significantly different between the different TIMP-1 concentrations and the control. Finally, *ALP* gene expression was significantly affected in rbASCs, with a significant increase at 100 ng/mL TIMP-1 compared to all the other conditions, while it showed a trend towards a dose–response in rbTenocytes, with higher *ALP* expression the higher the TIMP-1 concentration was compared to the control. This dose–response, however, turned out not to be statistically significantly different.

### 3.3. Wall Thickness, Fiber Thickness and Porosity of Electrospun Tubes

**Figure 3:**
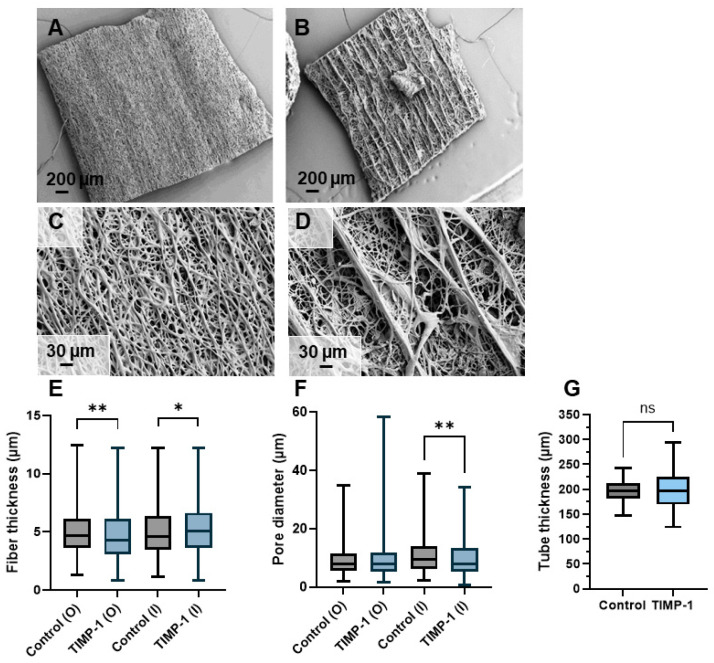
Characterization of novel TIMP-1 containing electrospun DegraPol^®^ meshes: outer surface of control (no TIMP-1) (**A**,**C**) and outer surface of DP with TIMP-1 (**B**,**D**); fiber diameters on outer (O) and inner (I) surfaces (**E**), porosity on outer (O) and inner (I) surfaces (**F**), and thickness of electrospun tubes (**G**). Key: Control = pure DP fibers without TIMP-1; * *p* ≤ 0.05, ** *p* ≤ 0.01, and ns = not significant.

The electrospun scaffolds were imaged with SEM and the inner and the outer surfaces were compared with respect to their fiber thickness and pore size. The wall thickness of the electrospun tubes was assessed as well (Figure 3). While the outer surface of the electrospun DP mesh without TIMP-1 was rather smooth (Figure 3A), the outer surface of the emulsion electrospun mesh containing TIMP-1 exhibited clearly visible grooves, with prominent relatively thick fiber peaks alternating with quite deep valleys of thin fibers (Figure 3B)—a 3D structure that emerged only in emulsion electrospun meshes.

As for the fiber thickness, a significantly smaller thickness for TIMP-1 containing fibers on the outer surface was determined; however, it was vice versa on the inner surface (Figure 3C). The pore diameter was similar for both kinds of meshes on the outer surface. For the inner surface, the pore diameter was smaller for the emulsion electrospun mesh containing TIMP-1 than for the control mesh without TIMP-1 (Figure 3D). Fiber thickness and pore diameter were assessed in electrospun tubes having very similar wall thicknesses (Figure 3E).

### 3.4. Water Contact Angles

**Figure 4:**
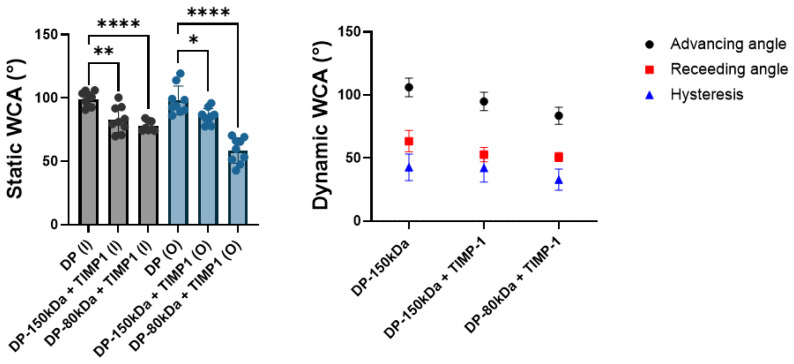
Showing (**A**) static WCA in degrees (°) for inner (I; black bars) and outer (O; blue bars) surfaces and for pure DP fibers (DP) as well as two different M_w_ DP emulsion electrospun meshes, with 150 kDa and 80 kDa, respectively, and (**B**) dynamic WCA in degrees (°), with advancing and receding angles as well as the hysteresis = advancing minus receding angle. Key: * *p* ≤ 0.05, ** *p* ≤ 0.01, and **** *p* ≤ 0.0001.

The static and dynamic water contact angles were assessed for pristine 150 kDa DP and for two emulsion electrospun DP meshes containing TIMP-1 in either 150 kDa or 80 kDa DP tubes, respectively (Figure 4). As expected, for the water-in-oil emulsion electrospun meshes, the contact angles were lower, indicating a more hydrophilic surface than for the pure DP fibers. This hydrophilic nature of emulsion electrospun meshes was even more prominent for the 80 kDa DP/TIMP-1 meshes compared with the 150 kDa DP/TIMP-1 meshes. The same was found for the dynamic WCA, with lower advancing and receding WCA for the emulsion electrospun meshes compared with the pure DP meshes, and lower angles for the 80 kDa compared with the 150 kDa DP/TIMP-1 fibers.

### 3.5. FTIR Spectra of Novel Electrospun Meshes

**Figure 5:**
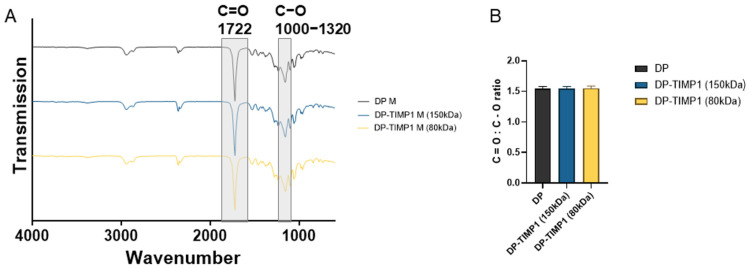
Averaged typical FTIR spectra of pure 150 kDa DP (black) and of TIMP-1 in either 150 kDa (blue) or 80 kDa DP (yellow) (**A**). The ratio of the C==O bond peak to the C−O bond peak for the three kinds of mesh (**B**). For the FTIR spectra of electrospun PEG and for each tube separately, please see Figure S1.

The novel electrospun DP fiber meshes containing TIMP-1 after emulsion electrospinning did not reveal different FITR spectra (Figure 5A). Also, the peak ratio of double C=O to single C−O bonds was not significantly different (Figure 5B), indicating the predominance of DP present over the protein TIMP-1, which added unobservable, negligible changes to the FTIR spectra.

### 3.6. TIMP-1 Release Kinetics

**Figure 6:**
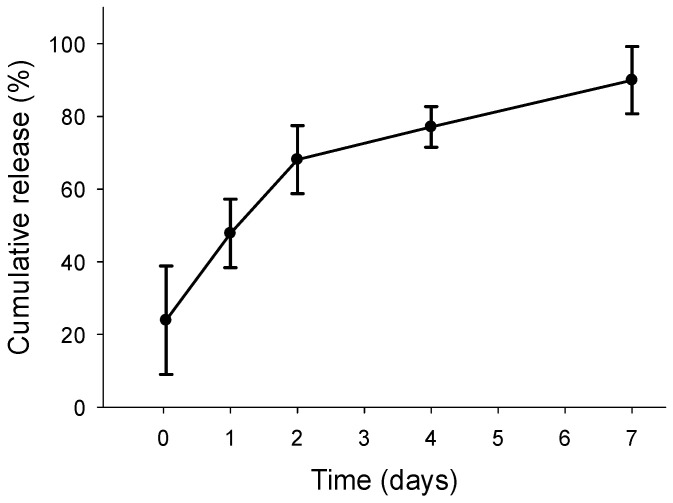
Characterization of the release kinetics of TIMP-1 from three 80 kDa DP tubes into a PBS + 0.1% RSA solution (mean of three tubes with three replicates and technical duplicates ± standard error of the mean). On the y-axis, 100% relates and corresponds to 1 ng TIMP-1 that has been released from a 0.5 cm long segment of the electrospun tube until day 7. Within such a segment, approximately 89 ng TIMP-1 have been incorporated, so 1 ng TIMP-1 corresponds to roughly 1.12% of the total TIMP-1 loading (1/89 = 0.0112). There was no statistically significant difference between the percentages at different time points.

The incorporation of TIMP-1 into the DP polymer fibers via emulsion electrospinning was verified by the assessment of the release kinetics into PBS with 0.1% RSA. Figure 6 shows an initial release of about 20% from the cumulative release at 7 days, followed by a slow and steady release up to one week. There was no statistically significant difference when the percentages of released TIMP-1 protein at different time points were compared.

## 4. Discussion

To study tendon healing biology [32], appropriate preclinical models are necessary [33,34] that allow an examination of a specific cytokine action under the complex environment in vivo [16]. Moreover, models that modulate the extracellular matrix (ECM) reconstitution in the healing tendon are of particular interest, because the recovered mechanical integrity caused by an optimum ECM composition is one of the crucial factors for patients suffering from tendon rupture; it decides on proper functionality and the time point to return to daily activities.

The rationale of the presented study was to select an important protein that regulates ECM regeneration in tendon healing and that may add to an increase in tendon strength at early time points post-laceration when sustainably delivered to the ruptured and sutured tendon. Besides ECM-degrading MMPs [18], their inhibitors, TIMPs, are gaining increasing attention due to their versatile function beyond metalloproteinase-dependent mechanisms [35]. TIMPs are able to interact with specific cell surface receptors and in turn activate signaling pathways in a metalloproteinase-independent way, affecting the proliferation, differentiation, angiogenesis, and apoptosis [36].

For several cell types, TIMP-1 has been reported to activate cell growth and proliferation, such as for keratinocytes, chondrocytes, epithelial cells, breast carcinoma cells, and fibroblasts [37,38,39]; however, it is not suitable for human bone marrow-derived mesenchymal stem cells (hBMSCs), where TIMP-1, in contrast, exhibited a negative regulatory role for the cell proliferation [40]. As tenocyte and mesenchymal stem cell proliferation may play pivotal roles during tendon regeneration in the early phase after acute injury, we assessed the proliferation of rbTenocytes and rbASCs with respect to TIMP-1 supplementation as a first step (Figure 1). The rabbit species was chosen as we intend to apply our new implant material that releases TIMP-1 in a rabbit Achilles tendon full-transection model in the future [34]. Although these cell types were significantly different in their natural proliferation capacity, with rbASCs proliferating significantly faster than rbTenocytes, resulting in significantly different percentages of cell number increase between day 1 and 3, there was no significant impact of TIMP-1 supplementation on the proliferation; in neither of these cell types and at none of the selected TIMP-1 concentrations, i.e., 1, 10, and 100 ng/mL, respectively.

As a next step, the effect of TIMP-1 supplementation on the gene expression of the most abundant collagen found in tendon tissue was explored: *Col1A1* (Figure 2A). As expected, *Col1A1* gene expression increased with increasing TIMP-1 concentrations in the rbTenocytes. According to Bae et al., human dermal fibroblasts cultivated in a spheroid model exhibited elevated TIMP-1 concentrations and simultaneously an ECM overproduction while MMP expression was decreased [41]. Interestingly, however, regarding *Col1A1* gene expression, there was no impact of TIMP-1 supplementation on rbASCs (Figure 2A).

In contrast to *Col1A1* gene expression where the two cell types responded differently, both rbTenocytes and rbASCs were similarly affected by TIMP-1 in *ki67* gene expression; there was an increase in *ki67* expression by trend for 1 and 100 ng/mL TIMP-1 when compared to the control (Figure 2B). This stands in contrast to the findings obtained by the Alamar Blue assay, where the proliferation was not affected by the addition of TIMP-1 to the cell culture. It has to be considered, however, that the gene expression increase in *ki67* was determined not continuously during the experiment, but only at the endpoint of a 3-day culture; hence, the dynamics of gene and protein expression may differ—and only later than that, i.e., after 3 days, the proliferation of the cells may be positively impacted by TIMP-1 supplementation. Noteworthy to mention that not only *ki67*, but most genes are transcribed in the episodic bursts of RNA synthesis, so gene expression and protein expression dynamics are different and heterogeneous by nature [42].

In our previous studies, where we determined the effect of PDGF-BB and ascorbic acid on gene expression of rabbit tenocytes, we found a significant increase in *ki67* gene expression on day 3 in the presence of 25 ng/mL PDGF-BB; however, no impact of 50 μg/mL ascorbic acid on *ki67* gene expression for a 3-day culture was observed [43]. Very similar to PDGF-BB, we have also measured the effect of IGF-1 on rabbit tenocyte *ki67* gene expression and found a significant increase at levels of 1 ng/mL IGF-1; however, with a decrease towards higher concentrations such as 10 ng/mL IGF-1 [15], the result was very similar as found for the TIMP-1 stimulation here, where there was a decline between 1 and 10 ng/mL TIMP-1 in *ki67* gene expression (Figure 2B).

A typical tendon marker is *tenomodulin*; it is responsible for tenocyte proliferation and tendon maturation [44], tenogenesis in mesenchymal stem cells (MSCs) [45], the prevention of fibrovascular scar formation during early tendon healing [46], and the regulation of ECM [47]. We found that TIMP-1 supplementation to rbTenocyte culture led to an increase in *tenomodulin* gene expression for 1 and 100 ng/mL TIMP-1 when compared to the control, but not for 10 ng/mL TIMP-1 (Figure 2C), a very similar pattern was observed for *ki67* gene expression (Figure 2B). Therefore, the TIMP-1 stimulation of rbTenocytes may have a positive impact on tendon healing if applied in a controlled delivery to the healing tendon at early time points post-laceration. As for the rbASCs, there was no substantial effect of TIMP-1 stimulation on *tenomodulin* gene expression, except for the highest concentration (100 ng/mL TIMP-1) where it was increased. It has been reported that ASCs are able to undergo tenogenic differentiation when exposed to tenogenic medium [48], but also with merely seeding ASCs on a PDMS substrate mimicking the shapes of tenocytes and leading to a topography-triggered differentiation—without further tenogenic medium supplementation [49]. Hence, our findings for the TIMP-1 supplementation of rbASC culture leading to a positive induction of *tenomodulin* in both cell types represents a promising finding and supports the idea to incorporate TIMP-1 into a drug delivery system for rabbit Achilles tendon repair.

Alkaline phosphatase (*ALP*) belongs to the early markers during osteogenesis [50]. Because TIMP-1 has been shown to trigger *ALP* induction at the tendon–bone enthesis [27], we determined its effect on rbTenocyte and rbASC culture. There was a trend toward a dose–response with increasing *ALP* expression at higher TIMP-1 concentrations for the tenocyte culture, although not statistically significant (Figure 2D). For the rbASC culture, however, we found a statistically higher *ALP* expression for the 100 ng/mL TIMP-1 supplementation when compared to each of the other experimental concentrations. This may be of particular interest if the envisioned implant material releasing TIMP-1 is positioned near the enthesis rather than around the mid-substance: at the tendon–bone interface, where MSCs from the *Karger* and heel fat pads could help regenerating not only the tendon part, but also the calcified fibrocartilage or even parts of the bone [1].

Depending on the cell type under view, however, TIMP-1 may also exhibit opposite effects regarding osteogenesis. For example, it has been reported that TIMP-1 negatively regulates the osteogenic differentiation of hBMSCs, with the attenuation of RUNX-2 [40], while TIMP-1 knockdown significantly increases *ALP* expression [40]. Therefore, our finding that rbASCs were positively affected by TIMP-1 with respect to *ALP* gene expression is a further promising finding, supporting the idea of a TIMP-1-delivering implant fabrication for tendon repair, particularly when the implant is positioned at the enthesis.

Therefore, we fabricated a novel implant material by emulsion electrospinning, where TIMP-1 protein was incorporated into DegraPol^®^ (DP) fibers, and compared it to corresponding electrospun meshes without TIMP-1 (control). DP is a biodegradable, biocompatible, pH-neutral co-block polymer that was proved to have no adverse effect on cellular response when applied as an electrospun tube in a rabbit Achilles tendon full-transection model [25], so it may serve as a beneficial drug delivery vehicle. While the TIMP-1 containing DP meshes exhibited visible grooves on the outer surface, for the control, the outer surface was quite flat (no grooves) (Figure 3A,B). This stands in accordance with previous findings for IGF-1 DP meshes [15]. These grooves are advantageous because after flipping the tube, the grooves face the repaired tendons and help keep the implant in place (the rough surface prevents the implant from gliding).

The assessment of the fiber thickness revealed smaller diameters on the outer surface compared to the control without TIMP-1, while opposite results were obtained for the inner surface facing the flat metal target (Figure 3C). Previous studies with incorporated PDGF-BB using a 12 wt% DP polymer solution for emulsion electrospinning confirm the lower fiber diameter for emulsion electrospun fibers compared to pure DP fibers [24], also for incorporated IGF-1 on the outer surface [15], but not the observed higher fiber diameter at the inner surface of TIMP-1 meshes, where for corresponding IGF-1 incorporated meshes no significant difference had been found for the fiber diameter [15]. As an explanation, it may be that jet stability was good at the start, but declined over time of emulsion electrospinning caused by water droplet aggregation within the water-in-oil emulsion, with increased and earlier jet splitting resulting in smaller fibers at the end of an electrospinning session—visible at the outside of the tube (outer surface). Otherwise, the tubes with TIMP-1 had very similar wall thickness as the tubes without TIMP-1 (Figure 3E), a prerequisite to compare their fiber thickness and pore size.

As anticipated, the static water contact angle (WCA) of the emulsion electrospun meshes was significantly lower than for the control (pure DP fibers), because the small water droplets that are incorporated in the polymer render the material hydrophilic (Figure 4). We also tested DP of different chain lengths and found that the hydrophilic nature of the emulsion electrospun mesh was even more pronounced for shorter polymer chain lengths (80 kDa) compared with larger 150 kDa DP by trend. From these findings, we conclude that shorter polymer chains may lead to a more homogeneous distribution of eventually smaller droplets than larger polymer chains of the same composition do, resulting in an overall more hydrophilic surface for the lower M_w_ polymer emulsion electrospun meshes. Likewise, the dynamic WCAs were determined to get smaller in the order pure 150 kDa DP, emulsion electrospun 150 kDa DP, and emulsion electrospun 80 kDa DP, respectively, indicating an increase in hydrophilicity in this order. The hysteresis values were quite high for all the tested meshes, suggesting a rather heterogeneous and rough surface caused by the randomly distributed fibers exposed to the surface, very similar to previous findings [15,24].

To further characterize the novel implant materials, FTIR spectra were assessed (Figure 5 and Figure S1). As expected, the small amount of TIMP-1 protein incorporated in either the 150 kDa DP or the 80 kDa DP did not change the FTIR spectrum significantly when compared with the corresponding spectrum of pure DP. As a quantitative readout, we assessed the ratio of peak heights for the CO double-to-single bonds and found a constant ratio over the three experimental groups, resulting in a value of approximately 1.5 for all of the fibers.

Finally, the release kinetics of TIMP-1 protein from emulsion electrospun DP meshes were determined (Figure 6). It was found that TIMP-1 release exhibited a sustained release over seven days, although only to a low percentage of total TIMP-1 loading, with roughly 1 ng released from a total of approximately 89 ng TIMP-1 loading per piece of tube, which equals to ~1.1 % of the total TIMP-1 loading. Compared to other proteins, such as PDGF-BB, this was a slightly higher percentage of released protein after 1 week, because in the same time period, 0.7% of total PDGF-BB loading had been released from emulsion electrospun DP fibers [24], although the molecular weights are similar for the two proteins, with 24.3 kDa for PDGF-BB and 28 kDa for TIMP-1, respectively. The smaller protein IGF-1 with a molecular weight of 7.6 kDa, however, showed a release range of 3–15% from the total loading in DP fibers, which is higher compared to TIMP-1 release within 7 days [15]. As the diffusion of proteins out from the small water droplets within the DP polymer fiber depends on the size of the proteins, with greater size going along with a potentially slower release, these observed relative release fractions after 1 week can be explained with the corresponding protein sizes.

If one compares the TIMP-1 concentration released after 7 days (Figure 6) with the gene expression data (Figure 2), 1 ng/mL TIMP-1 would have an effect in *Col1A1*, *ki67*, and *tenomodulin* gene expression with a substantial increase only in rbTenocytes, but not in rbASCs. It has to be emphasized, however, that in vivo release kinetics can be assumed to be different from in vitro release kinetics. In vivo, the implant material faces a microenvironment with different enzymes, such as lipase that degrades the DegraPol^®^ polymer fibers by hydrolysis [51], potentially leading to a faster TIMP-1 release from the mesh than observed under in vitro conditions with PBS + 0.1% RSA as release medium.

Limitations: For the Alamar Blue-assisted proliferation assessment, longer periods of time than 3 days are missing so far. It would be interesting to investigate if the impact of TIMP-1 that was found for *ki67* gene expression for both cell types, rbTenocytes and rbASCs, after 3 days, would also have an impact on the cell proliferation at time points later than 3 days, as *ki67* protein expression might be increased only at later time points than 3 days caused by different gene and protein expression dynamics.

## 5. Conclusions

Regulating ECM remodeling in preclinical animal models of tendon rupture repair demand for implant materials that affect the ECM remodeling and composition. We have chosen the protein TIMP-1, an inhibitor of MMPs and proliferation- and anti-apoptosis-promoting cytokine, and incorporated it into DP random fiber meshes by emulsion electrospinning for the first time. Based on TIMP-1 impact on the gene expression of rbTenocytes and rbASCs, where TIMP-1 increased *Col1A1* gene expression in rbTenocytes, *ki67* gene expression in both cell types, *tenomodulin* in both cell types at high concentrations (100 ng/mL), and *ALP* gene expression in rbASCs, we judge our novel elastic electrospun tube that releases TIMP-1 in a controlled slow way as a suitable implant material to be tested in the rabbit full-transection model to obtain insight into its in vivo function—particularly when positioned at the bone–tendon enthesis—and potentially pave the way for subsequent clinical trials.

## Data Availability

Data are available upon request.

## Supplementary Materials

The following supporting information can be downloaded at: https://www.mdpi.com/article/10.3390/ma18030665/s1. Figure S1: Further FTIR spectra, including the spectrum of pure electrospun PEG for comparison (PEG-spinned). For better comparability, the spectra are grouped in pure DP (DP 1, 2, and 3) and emulsion electrospun TIMP-1 tubes (DP-TIMP1-tube 1–3) for either 150 kDa or 80 kDa, respectively. Table S1: Primer sequences (rabbit). Genes, the NCBI reference sequence (used as a template for primer design), amplicon size, and forward and reverse primer sequences used for the qPCR analysis.
