# Supplementary material for: Fabrication and Characterization of Electrospun DegraPol® Tubes Releasing TIMP-1 Protein to Modulate Tendon Healing
Source: Materials (Basel). 2025 Feb 3;18(3):665. doi: 10.3390/ma18030665 (PMC11820012; doi:10.3390/ma18030665)
Supplement: Supplementary file 1 [file materials-18-00665-s001.zip › materials-3411678-supplementary.pdf]

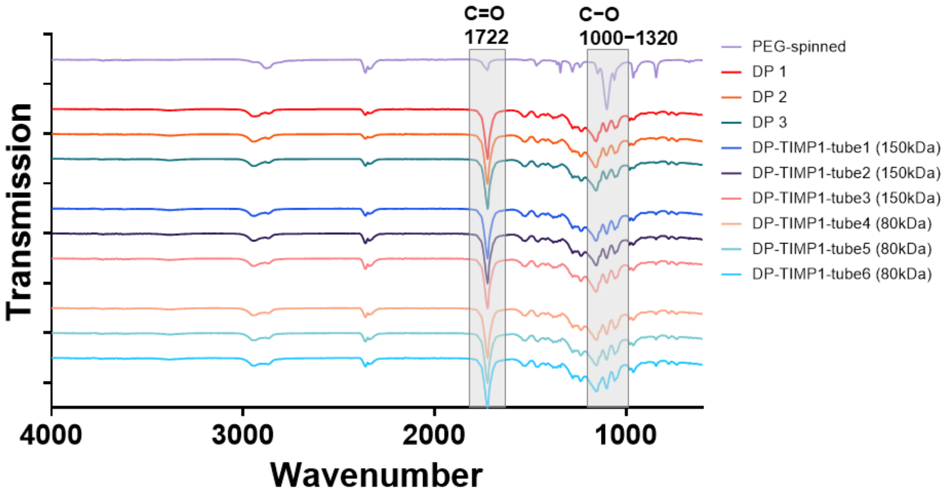

Figure S1 Further FTIR spectra, including the spectrum of pure electrospun PEG for comparison (PEG-spinned). For better comparability, the spectra are grouped in pure DP (DP 1, 2 and 3) and emulsion electrospun TIMP-1 tubes (DP-TIMP1-tube 1-3) for either 150 kDa and 80 kDa, respectively.

Table S1: Primer sequences (rabbit). Genes, the NCBI reference sequence (used as template for primer design), amplicon size and forward and reverse primer sequences used for qPCR analysis.

Gene	mRNA ID (NCBI Reference Sequence)	Amplicon size (bp)	Forward Primer Sequence (3′ → 5′)	Reverse Primer Sequence (5′ → 3′)
18S	NR_033238.1	176	GGAACTGAGGCCAT GATTAAG	CGGAACTACGACGG TATCTG
Col1A1	XM_008271783.1	271	CTGGTGAATCTGGA CGTGAG	TGTCTCACCCTTGTC ACCAC
Ki67	XM_008251084.2	283	CACATCCAGCAGTG AAACGG	GTGTTAGCAGTACC TGAAGTC
Tenomodulin	NM_001109818.1	239	GCAGTTTCCGAGTT ACAAGAC	CGACGGCAGTAAAT ACAACAG
ALP	XM_017346489	76	GTTCTCCATCAGGT GTCA	GGTCAGTGATGTTG TTCC
